# Quality and Readability of Arabic Online Resources on Early Orthodontic Intervention in Children: An Infodemiologic Study

**DOI:** 10.7759/cureus.100518

**Published:** 2025-12-31

**Authors:** Aliyah Aloraini, Deem Aljulaydan, Ghada Alaglan

**Affiliations:** 1 Department of Dentistry, College of Dentistry, Qassim University, Buraidah, SAU; 2 Department of Orthodontics and Pediatric Dentistry, College of Dentistry, Qassim University, Buraidah, SAU

**Keywords:** arabic websites, discern, early orthodontic treatment, honcode, jama benchmarks, online health information, orthodontics, quality assessment, readability

## Abstract

Background

Early orthodontic intervention during the primary or mixed dentition phase can effectively address malocclusion, mitigate detrimental oral habits, and enhance long-term oral and psychosocial outcomes. Although the American Association of Orthodontists recommends initiating orthodontic assessments for children at age seven, most caregivers consult the internet for information. The quality, reliability, and readability of Arabic-language internet sources regarding early orthodontic treatment remain deficient. This study aimed to assess Arabic websites on early orthodontic treatment in children using validated quality and readability instruments.

Methods

In October 2024, a Google search (Google Inc., Mountain View, CA, USA) was conducted using four Arabic search terms equivalent to “early orthodontic treatment in children.” The eligible websites were classified into different categories based on specialization, organizational affiliation, material type, and presentation style. Website quality was assessed using the DISCERN instrument, the Journal of the American Medical Association (JAMA) benchmarks, and the Health on the Net (HON) Code. Readability was assessed using the Flesch Reading Ease Score (FRES), Flesch-Kincaid Grade Level (FKGL), and Simplified Measure of Gobbledygook (SMOG) indices. Analysis of variance (ANOVA) and chi-square tests were used for statistical analysis, with p ≤ 0.05 considered statistically significant.

Results

The initial 200 search results were screened, resulting in 63 eligible sites. The DISCERN assessment indicated that Arabic-language websites concerning early orthodontic treatment exhibited moderate overall quality. The findings from the Journal of the American Medical Association (JAMA) benchmark analysis reinforce these conclusions, with only six (9.5%) websites meeting all four items. None of the included websites was HONcode-certified. University-affiliated websites achieved significantly higher DISCERN and JAMA benchmark scores than commercial or nonprofit websites (p ≤ 0.05). All websites demonstrated high readability (mean FRES > 80), with university-affiliated content written at the most accessible level (p = 0.001).

Conclusions

Arabic websites on early orthodontic treatment generally display high readability and moderate informational quality. Nonetheless, major gaps remain in authorship transparency, source citations, and the disclosure of conflicts of interest. Increasing government participation and standardizing transparency practices are essential to ensure reliable, evidence-based Arabic orthodontic content for caregivers and patients.

## Introduction

Orthodontic issues are common in children and can have long-lasting effects on their oral and mental health if left untreated. Early orthodontic intervention aims to correct malocclusions occurring during the primary or early mixed dentition phases, prevent harmful oral habits, and guide the growth of the maxilla and mandible before the eruption of the full permanent dentition [1]. An earlier diagnosis of orthodontic problems allows doctors to reduce future treatment complexity and complications, shorten the duration of treatment, and prevent more invasive procedures, such as tooth extraction or orthognathic surgery [2-4].

The American Association of Orthodontists recommends that children receive their first orthodontic evaluation by age seven. Usually, enough permanent teeth have erupted for a trained orthodontist to detect early signs of jaw problems, crowding, crossbites, or teeth emerging in abnormal positions. Finding these problems early provides an opportunity to intervene before they worsen, which often leads to better and more stable long-term results.

Many parents and caregivers use the internet as their main source of health information, despite professional recommendations. More than 70% of adults now use websites such as Google, YouTube, and other health-related platforms to seek medical advice, including dental and orthodontic care [5]. The appeal of the internet comes from its accessibility, immediacy, and wide variety of content. However, web-based information can differ significantly in terms of quality, accuracy, and readability. Many websites present incomplete, outdated, or biased information that may mislead users or contradict evidence-based clinical guidelines [6,7]. Recent studies have further demonstrated that misleading dental information is common online and actively engaged with by users, underscoring the importance of evaluating the quality of web-based oral health resources [8].

Readability is another critical factor determining the relevance of online health information. People with low health literacy have greater difficulty understanding resources when the text is too technical for the general population. The data suggest that most online health articles, including those on dental and orthodontic conditions, exceed recommended reading levels, making them difficult for the average person to understand [9].

Many infodemiological studies have evaluated the quality and readability of English-language internet content related to dental topics, such as orthodontic pain, orthognathic surgery, and clear alignment techniques. However, very little has been reported on Arabic-language resources that focus on early orthodontic interventions. More than 400 million people speak Arabic globally, and Arabic is the official language of more than 20 countries. This gap is a major global public health concern. Without adequate Arabic-language health information, people may be misled, delay treatment, or fail to follow professional orthodontic recommendations effectively.

Therefore, this study aimed to deliver a detailed infodemiologic analysis of Arabic-language online resources for early orthodontic intervention in children. More precisely, we assessed these tools based on the following criteria: (1) Quality and reliability: assessed using standardized tools, including the DISCERN instrument and Journal of the American Medical Association (JAMA) benchmarks. (2) Readability: evaluated using the Flesch Reading Ease Score (FRES), Flesch-Kincaid Grade Level (FKGL), and Simplified Measure of Gobbledygook (SMOG).

By reviewing these parameters, this study aimed not only to highlight the limitations of existing Arabic digital health resources but also to provide a baseline for developing more accurate, accessible, and patient-oriented content. Ultimately, the findings aim to help Arabic-speaking parents and caregivers make informed decisions and support healthcare practitioners in developing reliable, evidence-based online educational materials.

## Materials and methods

Search strategy

A systematic online search was conducted in October 2024 using Google (Google Inc., Mountain View, CA, USA) because it has the largest market share among Arabic-language users. The Google search was conducted in Chrome Incognito mode, with cookies and browser data cleared to ensure a bias-free approach. Four Arabic search phrases equivalent to "early orthodontic treatment in children" were used. The first 50 websites from each search phase were screened for eligibility. Typical user behaviors were simulated, resulting in a total of 200 websites screened. Duplicates were checked and, when present, were removed. To ensure relevant websites were included, specific eligibility criteria were applied to exclude irrelevant websites, sites containing only brief or superficial mentions of early orthodontics, scientific journal articles, news outlets, social media, blogs, forums, audio- or video-only content, websites with banner ads or sponsored links, and sites requiring login or subscription access. Then, the relevant websites presenting health information about early orthodontic treatment in the Arabic language that were freely accessible were included and evaluated for quality and readability.

Website classification

Eligible websites were classified using the framework proposed by Riordain and McCreary [10], based on specialization (content exclusively or partially focused on early orthodontics), organizational affiliation (commercial, nonprofit, governmental, or university/medical center), type of content (medical facts, clinical trials, questions and answers, or human interest stories), and presentation style (image, video, or audio).

Quality and reliability assessment

The included websites were reviewed using three markers.

DISCERN Instrument

The DISCERN Instrument [11] is a standardized 16-question tool used to evaluate the quality of health-related information. The first section (questions 1-8) assesses the publication's credibility to determine its reliability as a source of information on a specific therapy. The second section (questions 9-15) focuses on treatment alternatives. Question 16 represents the overall quality score of the evaluation. A five-point Likert scale was employed to evaluate each question, with 1 denoting poor quality and 5 signifying excellent quality. The total scores were categorized as low (16-32), moderate (33-64), or high (65-80).

JAMA Benchmark

The JAMA benchmark [12] evaluates four criteria-authorship (identification of the content authors, including their affiliations and relevant credentials), attribution (sources of information, including references or studies), currency (clear indication of when the content was posted or updated), and disclosure (ownership and declaration of any conflicts of interest).

Health on the Net (HON) Code Certification 

Websites are evaluated according to the HONcode criteria [13]. The HON Foundation assesses health-related websites based on eight standards for superior quality and transparent data disclosure. Websites meeting these criteria receive an HON seal for one year, requiring annual reassessments for renewal.

Readability assessment

Readability was assessed using a readability calculator based on three indices: FRES [14] scores range from 0 (very difficult) to 100 (very easy), with scores ≥ 80 considered acceptable; FKGL [15] indicates the U.S. school grade level, with scores < 7 considered readable, and SMOG [16] measures the complexity of medical text, with scores < 7 indicating adequate readability. With the presence of other readability tests, these are the applicable tests for the Arabic language based on multiple previous studies conducted to evaluate Arabic language content [17-19].

Rater training and calibration

Before data collection, two calibrated evaluators with orthodontic backgrounds and Arabic fluency (referred to as DA and AA) participated in a controlled calibration exercise overseen by an orthodontic specialist, during which a standardized scoring guide was developed and refined. Discrepancies were resolved to ensure a consistent interpretation of the rating criteria before the official assessment.

Inter-examiner reliability assessment

The inter-examiner reliability demonstrated excellent agreement between the two assessors. The intraclass correlation coefficient for the DISCERN total score was 0.97, indicating excellent reliability. The agreement for categorical JAMA benchmark items was excellent, with a Cohen’s kappa coefficient of 0.98.

Readability metrics, including the FRES, FKGL, and SMOG, were computed using standardized automated algorithms applied consistently to all included texts. As these metrics are algorithmic and produce consistent results when applied to identical information, inter-examiner reliability testing is unnecessary for readability assessment.

Data analysis

Data were collected in Microsoft Excel (Microsoft Corp., Redmond, WA, USA) and analyzed using IBM SPSS® statistics software (IBM Corp., Armonk, NY, USA). Normality was assessed using the Kolmogorov-Smirnov test. Normally distributed data were presented as mean ± standard deviation, whereas non-normally distributed data were reported as median and interquartile range. Analysis of variance (ANOVA) was used to compare continuous variables across the three groups. The chi-squared test was applied for categorical variables. To determine statistical significance in comparative tests, a P-value of ≤ 0.05 was considered significant.

Ethical considerations

Since the study examined publicly accessible online content without human participants or identifiable personal data, ethical approval or informed consent were not required.

## Results

Included websites and categorization

**Figure 1:**
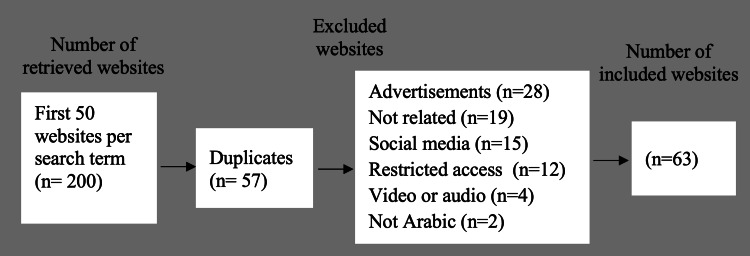
Flowchart of the search strategy

A total of 200 Arabic-language websites related to early orthodontic therapy were identified through a systematic Google search. Following the application of the inclusion and exclusion criteria, 63 websites were deemed eligible. The excluded websites included the following categories: 57 duplicates, 28 containing advertisements, 19 providing irrelevant information or brief mentions, 15 social media platforms, 12 with restricted access, four featuring only video or audio content, and two not in the Arabic language. Figure 1 shows a summary of our search strategy.

Most of the 63 Arabic-language websites reviewed (73.02%) were associated with universities or medical centers, whereas commercial and nonprofit organizations accounted for 14.29% and 12.70%, respectively. Importantly, none of the websites was associated with government bodies. Regarding specialization, 69.84% of the websites focused solely on early orthodontic treatment, whereas 30.16% covered it incompletely. Regarding content types, most websites contained medical information (71.43%), with smaller proportions presenting question-and-answer formats (23.81%), clinical trial details (3.17%), or human-interest stories (1.59%). Most content was presented visually, with 88.89% incorporating images, whereas videos (9.52%) and audio content (1.59%) were rarely included. None of the included websites was HONcode-certified, indicating substandard delivery of ethical and high-quality health information. Table 1 summarizes the categorization of the included websites according to affiliation, specialization, content type, presentation, and HONcode certification.

**Table 1:** Distribution of included websites on early orthodontic treatment by affiliation, specialization, content type, presentation format, and HONcode certification. Values are presented as frequencies (n) and percentages(%). HONcode: Health on the Net Code

Parameter		Frequency	Percentage
Affiliation	Commercial	9	14.29%
	Non-profit organization	8	12.70%
	University / medical center	46	73.02%
	Governmental	0	0.0%
Specialization	Exclusively related	44	69.84%
	partly related	19	30.16%
Content type	Medical facts	45	71.43%
	Clinical trial	2	3.17%
	Human interest stories	1	1.59%
	Question and answer	15	23.81%
Content presentation	Image	56	88.89%
	Video	6	9.52%
	Audio	1	1.59%
HONcode	Certified	0	0.0%
	Not certified	63	100.0%
Total		63	100.0%

Quality assessment

The DISCERN analysis showed that the quality of the assessed websites varied substantially across domains. In the reliability domain, items such as Q1 (explicit aims) and Q2 (aims achieved) scored moderately high, with mean values of 4.25 and 3.95, respectively. However, the transparency markers were weak, as Q4 (explicit sources) and Q7 (additional sources) had low mean scores of 1.68 and 2.06, respectively. The existence of publication and update dates (Q5) was also suboptimal (mean = 2.95). In the domain of treatment options, websites excelled in communicating Q9 (how treatment works; mean = 3.94) and Q10 (benefits of treatment; mean = 4.41), but performed moderately on Q11 (risk of treatment; mean = 3.27), Q12 (effects of no treatment; mean = 3.75), and Q15 (shared decision-making; mean = 3.13). The mean total DISCERN score (items Q1-Q15) across all included websites was 51.2 out of a possible 75, indicating moderate overall quality. The mean of the reliability section (items Q1-Q8) was 25.42, similar to the mean of the treatment evaluation section (items Q9-Q15), which had a mean of 25.76. A summary of the DISCERN analysis, including the means and medians of each question, along with the maximum and minimum scores, is presented in Table 2.

**Table 2:** DISCERN evaluation for the included websites Values are presented as mean, median with interquartile range (IQR), maximum (Max), and minimum (Min).

Domain	DISCERN question	Mean	Median	Std. Deviation	Minimum	Maximum
Reliability	Q1. Explicit aims	4.25	5	1.164	1	5
	Q2. Aims achieved	3.95	4	1.275	1	5
	Q3. Relevance	3.98	4	1.211	1	5
	Q4. Explicit sources	1.68	1	1.202	1	5
	Q5. Explicit date	2.95	3	1.708	1	5
	Q6. Balanced and unbiased	3.65	4	1.259	1	5
	Q7. Additional sources	2.06	1	1.33	1	5
	Q8. Areas of uncertainty	2.9	3	0.928	1	5
Treatment options	Q9 How treatment works	3.94	5	1.294	1	5
	Q10. Benefits of treatment	4.41	5	1.2	1	5
	Q11. Risk of treatment	3.27	3	1.558	1	5
	Q12. Effects of no treatment	3.75	4	1.47	1	5
	Q13. Effects on quality of life	3.63	4	1.451	1	5
	Q14. All alternatives described	3.63	4	1.371	1	5
	Q15. Shared decision	3.13	3	1.301	1	5
Overall rating	Q16. Overall quality rating	3.6	4	1.251	1	5

The assessment of overall quality using the DISCERN tool indicated that 47.6% of the websites exhibited high quality, with most (41.3%) affiliated with universities or medical centers. Among the assessed websites, 39.7% were of moderate quality, with 27.0% of these belonging to the same university/medical center category. Only 12.7% of the websites were classified as low quality, and this group was nearly evenly distributed across all affiliations, including commercial (4.8%), nonprofit (3.2%), and university-based sites (4.8%). The differences in DISCERN scores across affiliations were statistically significant (p = 0.050), as shown in Table 3.

**Table 3:** DISCERN quality categories distribution of the included websites based on their affiliation. Values are presented as frequencies (n) and percentages (%). Differences in DISCERN quality categories across websites based on their affiliations were assessed using the chi-square (χ²) test of independence; a p-value of ≤ 0.05 was considered significant. The total DISCERN scores were categorized as low (16-32), moderate (33-64), or high (65-80).

DISCERN score	Commercial	Non-profit organization	University/Medical center	Total	χ² (df)	p-value
Low	3 (4.8%)	2 (3.2%)	3 (4.8%)	8 (12.7%)	9.51(4)	0.050
Moderate	5 (7.9%)	3 (4.8%)	17 (27.0%)	25 (39.7%)		
High	1 (1.6%)	3 (4.8%)	26 (41.3%)	30 (47.6%)		
Total	9 (14.3%)	8 (12.7%)	46 (73.0%)	63 (100.0%)		

Assessment of the JAMA benchmark criteria revealed variable adherence across websites based on organizational affiliation. Of the four JAMA items, authorship was the most fulfilled criterion, met by 54.0% of the websites, predominantly from university- or medical center-affiliated websites (36.5%). Overall, attribution was met by 57.1% of the sites, with university-affiliated websites comprising 49.2%, indicating a statistically significant association with attribution (p = 0.007). Currency, which refers to the presence of date information, was satisfied by 69.8% of the websites, mostly university sites (54.0%), although this difference was not statistically significant (p = 0.387). Disclosure was the least fulfilled criterion overall (39.7%), with significant variation by affiliation (p = 0.027), as no commercial sites disclosed ownership or conflicts of interest, as shown in Table 4. 

**Table 4:** Distribution of JAMA benchmark criteria and number of items met by included websites based on their affiliation. JAMA: Journal of American Medical Association. Values are presented as frequencies (n) and percentages (%). Differences in the number of JAMA benchmark criteria met across included websites based on their affiliations were assessed using the chi-square (χ²) test of independence; a p-value of ≤ 0.05 was considered significant.

JAMA items		Commercial	Non-profit organization	University/Medical center	Total	χ² (df)	p-value
Authorship		7 (11.1%)	4 (6.3%)	23 (36.5%)	34 (54.0%)	2.39(2)	0.302
Attribution		1 (1.6%)	4 (6.3%)	31 (49.2%)	36 (57.1%)	9.86(2)	0.007
Currency		6 (9.5%)	4 (6.3%)	34 (54.0%)	44 (69.8%)	1.90(2)	0.387
Disclosure		0 (0.0%)	3 (4.8%)	22 (34.9%)	25 (39.7%)	7.20(2)	0.027
	JAMA benchmark number of items met						
Zero		2 (3.2%)	0 (0.0%)	6 (9.5%)	8 (12.7%)	13.37(8)	0.131
One		2 (3.2%)	4 (6.3%)	12 (19.0%)	18 (28.6%)		
Two		5 (7.9%)	0 (0.0%)	17 (27.0%)	22 (34.9%)		
Three		0 (0.0%)	3 (4.8%)	6 (9.5%)	9 (14.3%)		
Four		0 (0.0%)	1 (1.6%)	5 (7.9%)	6 (9.5%)		

When analyzing the number of JAMA criteria met per website, only 9.5% of websites met all four benchmarks, and 14.3% met three. The largest group (34.9%) satisfied two criteria, whereas 12.7% met none. The number of items achieved per website is presented in Table 4.

Readability assessment

All 63 analyzed websites achieved a FRES exceeding 80. None of the websites scored below this threshold. The highest mean FRES was observed for websites affiliated with universities or medical institutions (110.3 ± 4.28), closely followed by nonprofit organizations (109.8 ± 6.23) and commercial websites (105.0 ± 6.60). The difference in FRES scores between included websites based on their affiliations was statistically significant (p = 0.022; Table 5).

**Table 5:** FRES scores of the included websites based on their affiliation FRES: Flesch Reading Ease Scale Values are presented as frequencies (n) and percentages (%) or mean ± standard deviation. Differences in FRES mean scores across included websites based on their affiliations were assessed using one-way analysis of variance (ANOVA). A p-value of ≤ 0.05 was considered statistically significant.

FRES score	Commercial	Non-profit organization	University/Medical center	Total	F(df)	p-value
< 80	0 (0.0%)	0 (0.0%)	0 (0.0%)	0 (0.0%)	4.45(2, 60)	0.022
> 80	9 (14.3%)	8 (12.7%)	46 (73.0%)	63 (100.0%)		
Mean score (±SD)	105.0 ± 6.60	109.8 ± 6.23	110.3 ± 4.28	109.3 ± 5.2		

All evaluated websites recorded FKGL scores below 7, indicating that the reading difficulty was appropriate for elementary to middle school levels. Websites associated with universities or medical institutions had the lowest mean FKGL score (1.14 ± 1.18), followed by nonprofit organizations (1.96 ± 1.55) and commercial websites (3.26 ± 1.87). The differences in FKGL scores across the included website based on their affiliation were statistically significant (p = .001; Table 6).

**Table 6:** FKGL scores of the included website based on their affiliation FKGL: Flesch-Kincaid Grade Level Values are presented as frequencies (n) and percentages (%) or mean ± standard deviation. Differences in FKGL mean scores across included website based on their affiliation were assessed using one-way analysis of variance (ANOVA). A p-value of ≤ 0.05 was considered statistically significant.

FKGL score	Commercial	Non-profit organization	University/Medical center	Total	F(df)	p-value
< 7	9 (14.3%)	8 (12.7%)	46 (73.0%)	63 (100.0%)	9.89 (2, 60)	0.001
> 7	0 (0.0%)	0 (0.0%)	0 (0.0%)	0 (0.0%)		
Mean score (± SD)	3.26 ± 1.87	1.96 ± 1.55	1.14 ± 1.18	1.55 ± 1.52		

All 63 websites scored below the SMOG threshold of 7, indicating that the content was written at a level comprehensible to readers with basic literacy skills. The mean SMOG score for university- or medical center-affiliated websites was slightly higher (2.19 ± 0.40) than that for nonprofit organizations (1.96 ± 0.24) and commercial websites (1.92 ± 0.99). The differences in SMOG scores among included websites based on their affiliations did not reach statistical significance (p = 0.066; Table 7).

**Table 7:** SMOG scores of the included websites based on their affiliation SMOG: Simple Measure of Gobbledygook Values are presented as number (percentage) or mean ± standard deviation. Differences in SMOG mean scores across included website based on their affiliations were assessed using one-way analysis of variance (ANOVA). A p-value of ≤ 0.05 was considered statistically significant.

SMOG score	Commercial	Non-profit organization	University/Medical center	Total	F(df)	p-value
< 7	9 (14.3%)	8 (12.7%)	46 (73.0%)	63 (100.0%)	2.78 (2, 60)	0.066
> 7	0 (0.0%)	0 (0.0%)	0 (0.0%)	0 (0.0%)		
Mean score (± SD)	1.92 ± 0.99	1.96 ± 0.24	2.19 ± 0.40	2.12 ± 0.38		

## Discussion

The present study is the first to systematically evaluate Arabic-language websites related to early orthodontic treatment in children using validated quality and readability tools. Google was chosen as the sole search engine for this study because it is the most commonly used search engine in Arabic-speaking communities and the predominant platform through which the public seeks online health information. Previous infodemiological studies assessing Arabic-language dental and medical content have also relied exclusively on Google searches, which account for most of internet search activity in the Middle East and North Africa [20]. Google is therefore considered an appropriate platform for a realistic portrayal of caregiver search behavior and maximizes the external validity of the findings. A comparable investigation conducted by Alpaydın et al., which assessed English-language content related to early orthodontic treatment, included 86 websites out of a pool of 200, similar to the present study. Both studies applied the same exclusion criteria, with most websites in the present study excluded because they consisted exclusively of advertisements without substantive content [6].

The DISCERN assessment indicated that Arabic-language websites concerning early orthodontic treatment exhibited moderate overall quality, with significant variation across domains. The clarity of purpose and elucidation of treatment were the primary strengths, as most websites effectively conveyed their objectives and provided sufficient information regarding the mechanisms and potential advantages of early orthodontic treatment. This suggests that content creators should prioritize explaining the clinical rationale and benefits of early interventions in ways that caregivers can understand. However, significant deficiencies were observed in the transparency-related elements of the DISCERN instrument. Poor adherence to evidence-based reporting standards was indicated by low scores in the disclosure of publication or update dates, supplementary references, and explicit source citations. Regarding the treatment evaluation domain, most websites excelled in explaining the benefits and mechanisms of treatment but showed only moderate performance in describing the risks of treatment, the consequences of no treatment, and shared decision-making. The lower emphasis on risks and alternative treatment options may indicate an inclination toward promotional and reassuring language rather than objective patient education. This could lead to unrealistic expectations among parents and caregivers regarding early orthodontic treatment outcomes and may also affect their informed decision-making.

Findings from the JAMA benchmark analysis support these conclusions, with only six websites achieving all four items; five of these websites were affiliated with universities or medical centers, and one website belonged to a nonprofit organization. This finding is similar to Alpaydin et al.’s study, which evaluated English-language content on early orthodontic treatment, in which only three websites met all JAMA criteria. While authorship, attribution, and currency were met by a substantial proportion of websites, disclosure of ownership or conflicts of interest was the least frequently satisfied criterion, in contrast to Alpaydin et al.’s study, in which disclosure was the most frequently met item [6].

All assessed websites lacked HONcode certification, indicating inadequate adherence to ethical and quality standards in the dissemination of health information, a finding that has also been reported in several other studies on Arabic dental content [21-23].

One significant strength noted was the exceptionally high readability of Arabic content, with all websites achieving FRES scores above 80, FKGL values at levels accessible to early-grade readers, and SMOG scores above 7, indicating that the content was written at a level suitable for readers with basic literacy skills. This finding aligns with the results of other Arabic web-based studies [17,18,21] and contrasts with English-language studies [6,9], in which online health content tended to exceed the recommended reading level. The relatively simple structure of the language used in Arabic health websites may imply a deliberate effort to target caregivers with varying levels of health literacy. Additionally, the diglossic nature of the Arabic language, in which Modern Standard Arabic is used for formal communication, may lead writers to deliberately simplify the text to ensure intelligibility across various regional dialects.

These findings align with previous research on web-based Arabic content, which has demonstrated that authors of online health websites often prioritize readability and audience engagement over documentation from scientific sources [17,18,21]. This deficiency reduces the perceived trustworthiness of the content and may mislead caregivers. This may be owing to the lack of robust regulatory monitoring of internet health materials in Arabic-speaking regions, where readability may be achieved at the expense of scientific integrity and transparency.

Our results showed that websites associated with universities or medical centers consistently outperformed other affiliations in terms of both quality (DISCERN and JAMA) and readability (FRES, FKGL, and SMOG). These results align with those of earlier English-language studies [6,7], which showed that academically affiliated sites performed better than commercial websites. Higher education institutions and physicians valued evidence-based knowledge and consistently scored highly on measures of comprehensiveness and scientific rigor [24].

Another concern is the lack of government websites, which are generally viewed as highly reliable sources. This has created a gap that warrants greater government involvement in developing standardized, evidence-based Arabic-language health portals.

This study had some limitations. First, the webpages retrieved in this study were sourced from a single search engine, unlike those in several other studies. Google has been the predominant search engine for most individuals worldwide over the past decade. Another important limitation is that the readability tests employed in this study were originally designed to evaluate English texts based on U.S. grade levels and may lack accuracy when applied to other languages, despite their use in prior research. Finally, this study treated Arabic websites as a homogeneous group, neglecting regional variation among Arabic speakers, which may affect the presentation and interpretation of digital health information. This may affect the generalizability of the results. Future studies should address these limitations comprehensively.

## Conclusions

This study presents the first epidemiological evaluation of Arabic-language online resources for early orthodontic treatment in children. The findings indicate that most websites have good readability and moderate quality, with key weaknesses in transparency, referencing, and disclosure practices. These deficiencies may undermine the reliability of online health information.

Efforts should be made to improve web-based Arabic health information by (1) increasing governmental involvement in developing and maintaining evidence-based Arabic health portals; (2) encouraging websites to adopt standardized transparency practices, including clear authorship, attribution, and disclosure; and (3) ensuring that readability remains accessible while improving the depth and accuracy of content. Overcoming these challenges may equip Arabic-speaking caregivers with the knowledge needed for effective decision-making regarding early orthodontic treatment for their children.
